# Spleen stiffness measurements during recompensation in patients with acutely decompensated liver cirrhosis: preliminary findings of a pilot study

**DOI:** 10.3389/fmed.2024.1475997

**Published:** 2024-11-25

**Authors:** Dilan Elcin Bozal, Jonathan Hannemann, Martin Bürger, Gabriel Allo, Eva Dittmann, Anna Martin, Natalie Jaspers, Bianca Holzapfel, Seung-Hun Chon, Sonja Lang, Tobias Goeser, Hans-Michael Steffen, Philipp Kasper

**Affiliations:** ^1^Department of Gastroenterology and Hepatology, Faculty of Medicine, University Hospital Cologne, University of Cologne, Cologne, Germany; ^2^Department of General, Visceral, Tumor, and Transplantation Surgery, Faculty of Medicine, University Hospital Cologne, University of Cologne, Cologne, Germany

**Keywords:** spleen stiffness, SSM, portal hypertension, decompensation, decompensated cirrhosis, CSPH, recompensation

## Abstract

**Background:**

Acute decompensation (AD) in patients with liver cirrhosis is associated with a dramatic deterioration in prognosis. Immediate initiation of appropriate recompensation measures is essential to improve patient’s outcome, although objective parameters for evaluating the success of recompensation are still lacking. Spleen stiffness measurements (SSM) have emerged as promising non-invasive tool to assess clinically significant portal hypertension (CSPH), which is the main driver of acute decompensation. However, while SSM accurately predicts CSPH and its complication, currently no data are available on its diagnostic performance during recompensation. This pilot-study aimed at evaluating changes in spleen stiffness following the initiation of recompensation measures in cirrhotic patients hospitalized due to AD.

**Methods:**

In this prospective pilot-study, 60 patients with cirrhosis showing AD were included. Liver stiffness measurements (LSM) and SSM were performed on admission and repetitive SSM on day 3 and 5, respectivele, during recompensation measures. A cohort of patients (*n* = 10) with compensated cirrhosis served as control.

**Results:**

A total of 36 data sets from the originally enrolled 60 patients were eligible for final analysis. On admission, patients with AD revealed a significantly increased spleen stiffness compared to the control group (70.51 vs. 29.06 kPa, *p* < 0.0001). Following the initiation of recompensation measures SSM revealed a significant reduction in spleen stiffness compared to the baseline assessment on day 3 (−18.5 kPa, −21.53%; *p* = 0.0002) with no further decrease on day 5 (−17.63 kPa, −21.23%; *p* = 0.0326).

**Conclusion:**

Repetitive SSM seems to be a useful non-invasive clinical marker to assess the effectiveness of recompensation measures in cirrhotic patients with AD.

## 1 Introduction

Liver cirrhosis represents the final stage of chronic liver diseases, such as chronic viral hepatitis, alcohol abuse or metabolic-dysfunction associated steatotic liver disease (MASLD), and is associated with high morbidity and mortality (1–3). The clinical course of liver cirrhosis can be divided into a compensated and a decompensated stage. Compensated cirrhosis is clinically defined as a stable stage of the disease characterized by absent or only minor symptoms (4, 5). Decompensated cirrhosis is defined by the manifestation of clinical complications, such as jaundice, ascites, portal-hypertensive bleeding, hepatic encephalopathy, or infections (3, 5, 6). The transition into a decompensated disease stage occurs at a rate of 4–12% per year and is linked to a considerably increased risk of experiencing further decompensating events, hospitalization and death, thus indicating a prognostic watershed in the clinical course of the disease (7, 8). After the first decompensation, further decompensating events occur in up to 60% of patients and mortality increases significantly (9). The key to improve the prognosis of patients with a decompensated cirrhosis is the rapid and complete recompensation with the aim to prevent further episodes of decompensation.

Recompensation is characterized by a resolution of liver-related complications, such as ascites, hepatic encephalopathy, or portal hypertensive bleeding along with an improvement of liver function. While a rapid and efficient recompensation appears to be essential, data on the assessment of hepatic recompensation are limited. The therapeutic success of recompensating measures is based primarily on the judgment of the treating physician and symptom assessment, while non-invasive biomarkers to monitor the recompensation process are lacking.

One factor that significantly influences the occurrence of decompensation, but also recompensation, is the severity of the underlying portal hypertension (10). Portal hypertension develops due to structural and functional changes in the cirrhotic liver and is a key driver of disease progression with its inherent risk of decompensation in patients with cirrhosis (10–13). Numerous studies have shown that a reduction of clinically significant portal hypertension (CSPH), for example with non-selective beta-blocker treatment, translates into a decreased risk of a first decompensation and adverse liver-related events, including repeated episodes of decompensation and liver-related mortality (10, 14, 15).

Different modalities are currently available to identify patients with CSPH that is defined as a hepatic venous pressure gradient (HVPG) ≥ 10 mmHg. However, while measurement of the hepatic venous pressure gradient is the gold standard for assessing CSPH, it represents an invasive procedure that is only available in experienced centers. Therefore, non-invasive tests, based on transient elastography, have become increasingly important to determine the severity of CSPH and its prognostic implications, as well as to guide treatment decisions (16, 17). In addition to liver stiffness measurement (LSM), spleen stiffness measurement (SSM) is also being carried out more frequently for risk prediction of acute decompensation, as SSM can detect early changes in portal hemodynamics (18–20).

SSM is an easily applicable vibration-controlled transient elastography technique, that allows real-time bedside assessment of CSPH (20–22).

Recent studies have demonstrated that SSM provides a valuable non-invasive surrogate marker of CSPH and is thought to outperform LSM in risk prediction for clinical decompensation (23).

However, whether and to what extend repetitive SSM may be helpful in the clinical assessment of recompensation rens unclear. Therefore, the aim of the present pilot study was to investigate the feasibility of repetitive SSM and its clinical usefulness after initiation of recompensation measures in patients with cirrhosis hospitalized due to acute decompensation.

## 2 Materials and methods

### 2.1 Study population and study design

For this prospective single-center pilot study, we included consecutive patients with cirrhosis after hospitalization due to acute decompensation at a tertiary care hospital (Department of Gastroenterology and Hepatology, University Hospital of Cologne, Germany) between 01/23 and 06/24, and performed LSM and repetitive SSM during recompensation. Acute decompensation of cirrhosis was defined as the development of ascites (>grade 1), overt hepatic encephalopathy, bacterial infection, hepatorenal syndrome, portal-hypertensive bleeding or a hepatic hydrothorax leading to hospital admission. Recompensation was defined as resolution of ascites and hepatic encephalopathy, absence of variceal re-bleeding, improvement of renal function parameter on laboratory analysis and control of infection. For recompensation, paracentesis was performed for ascites accompanied by adjustment of diuretics, administration of lactulose was performed for hepatic encephalopathy, administration of albumin and terlipressin for hepatorenal syndrome and endoscopic ligation together with non-selective beta blocker treatment in the case of variceal hemorrhage.

A group of patients with stable compensated cirrhosis, without any clinical signs of an acute decompensation, served as control group.

All patients with portal or mesenteric vein thrombosis, myeloproliferative diseases, previous abdominal surgery, transjugular intrahepatic portosystemic shunt, as well as patients with post-hepatic portal hypertension due to Budd-Chiari syndrome or cirrhose cardiaque were excluded from the study. Additional exclusion criteria were the presence of non-cirrhotic portal hypertensive diseases (e.g., hepatosplenic schistosomiasis, porto-sinusoidal vascular disease) as well as presence of a hepatocellular carcinoma or other hepatic malignancies, as well as failure of vibration-controlled transient elastography examinations or a lack of informed consent.

The presented study was conducted in accordance with the Declaration of Helsinki 1964 and its further amendments. Written informed consent was obtained by all study participants.

### 2.2 Liver and spleen stiffness measurements

Measurements of liver and spleen stiffness were performed by vibration-controlled transient elastography using the FibroScan^®^ 630 Expert device (Echosens, Paris, France) with a 50 Hz probe for the liver and a 100 Hz spleen-dedicated module coupled with an ultrasound localization system, respectively (23–26).

In detail, LSM and SSM were performed in overnight fasted patients in a supine position with the right and left arms, respectively, in maximum abduction and by placing the transducer in the intercostal spaces, as previously described (26). For the SSM, the 100 Hz probe was placed at an ultrasound-targeted localization, where the spleen parenchyma had been previously identified. Results were expressed in kPa and LSM and SSM values were considered reliable, if at least 10 successful measurements were obtained, with a success rate of at least 60%, and an interquartile range (IQR) to median ratio <30% (26, 27).

**Figure 1:**
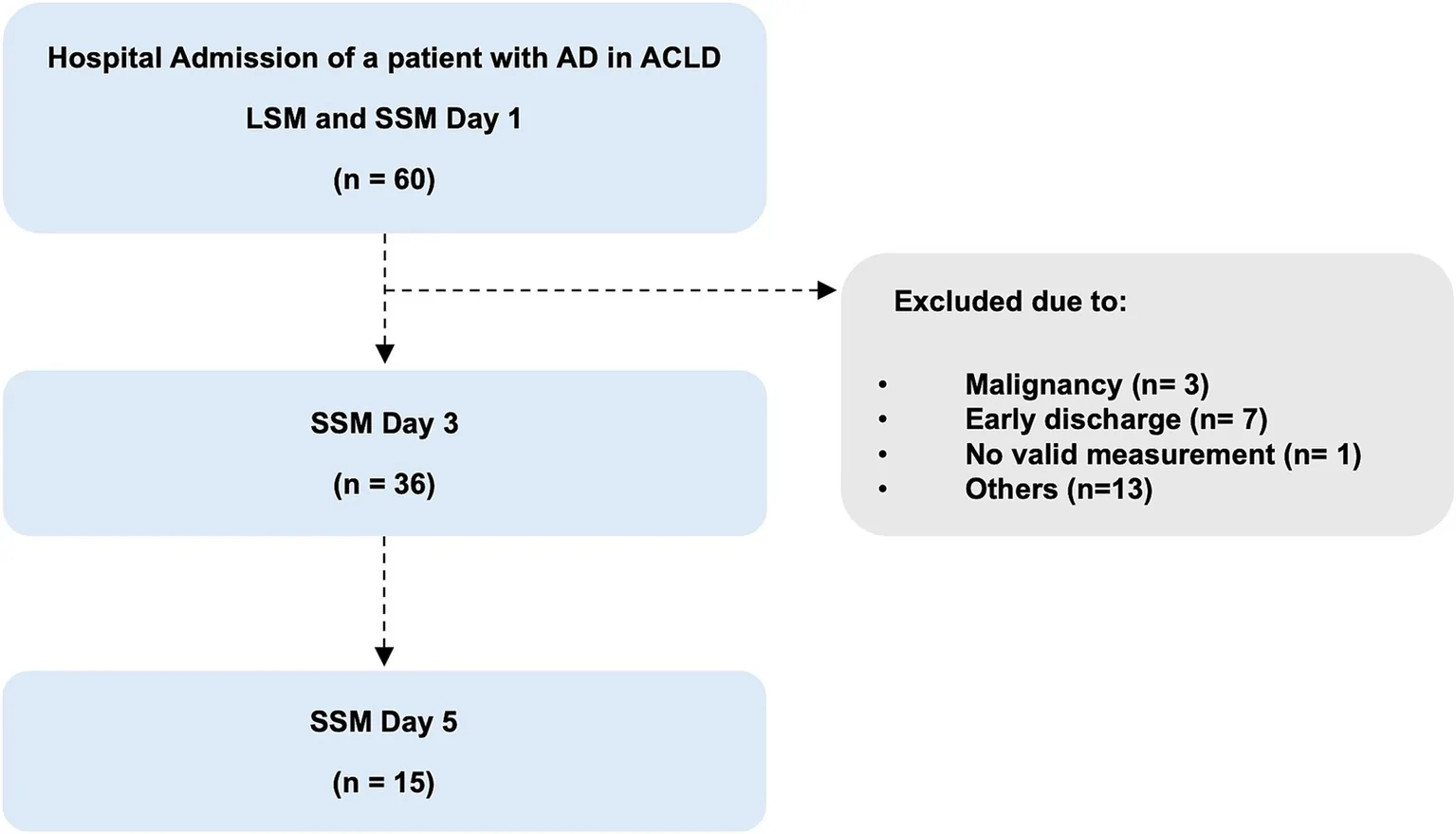
Flowchart of the study population. AD, acute decompensation; SSM, spleen stiffness measurement; LSM, liver stiffness measurement.

Within this pilot study, SSM were initially only performed during the short-term course of recompensation measures, after admission (day 1), at day three (day 3) and day five (day 5) after initiation of recompensation measures. LSM was also performed at admission, at day one. In order to compare the values of the acute decompensation group, both SSM and LSM were also performed in a control group of individuals with stable compensated cirrhosis. The patients in the control group were recruited from the outpatient liver center. Blood samples for laboratory analyses were collected under fasting conditions (Figure 1).

### 2.3 Statistical analysis

For baseline characteristics, continuous variables are presented as means and standard deviation (SD), while the categorical variables are presented as frequencies with percentages. To compare differences between two groups, Student’s *t*-test was used for continuous variables, while the Chi^2^-test was employed for categorical variables. Statistical analyses were carried out using GraphPad Prism, Version 10.2.3 (Boston, MA 02110, USA). A two-sided *p*-value 0.05 was considered as statistically significant.

## 3 Results

In total, 60 patients with liver cirrhosis admitted to the hospital due to acute decompensation were evaluated for eligibility. After checking the eligibility criteria, 24 patients were excluded for the following reasons: early discharge (*n* = 7), new diagnosis of malignancy (*n* = 3), withdrawal of initial consent and rejection of further measurements (*n* = 5), misclassification (*n* = 4), inability of spleen stiffness measurements due to anatomical (*n* = 1) or technical reasons (*n* = 4) (Figure 1).

Finally, 36 patients completed at least two or more measurements of spleen stiffness during recompensation. In detail, 15 of these 36 patients were measured at day 1, 3, and 5 and 21 patients were measured at day 1 and 3 (Figure 1).

The mean age of the patients with acute decompensation enrolled was 60.8 ± 9.9 years, with 52.8% (*n* = 19) of them being male. The primary causes of cirrhosis in the study cohort of patients with acute decompensation were alcohol-related liver disease (42.7%) followed by MASLD (22.2%). The mean age of the control group was 58.5 ± 3.7 years, while 40% were female and the most common cause of cirrhosis was MASLD (40%), followed by alcohol (30%) and viral (30%). The clinical events of decompensation included the development of ascites in 12 patients (33.3%), hepatic encephalopathy in eight patients (22.2%), bacterial infection in seven patients (19.4%), hepato-renal syndrome in four patients (11.1%), gastrointestinal portal hypertensive hemorrhage in four cirrhotic patients (11.1%) and the manifestation of a hepatic hydrothorax in one patient with cirrhosis (2.8%). Of the four patients with portal hypertensive gastrointestinal bleeding, three patients already received NSBB (carvedilol) as a primary prophylaxis due to CSPH with high-risk varices or due to previous episodes of variceal hemorrhage. In one patient, NSBB treatment was newly initiated after the portal-hypertensive bleeding episode. In all patients, NSBB treatment was continued as secondary prophylaxis. Carvedilol was always used, and the treatment effectiveness was evaluated based on the change in heart rate.

On admission, the majority of decompensated patients presented with a Child-Pugh class B (52.8%), while 14 patients (38.9%) had a Child-Pugh class C and three patients with acute decompensation (8.3%) presented to the hospital with a Child-Pugh class A. All patients of the control group were in Child-Pugh class A.

At baseline, the mean Model for End-Stage Liver Disease (MELD) score of the study group was 16, compared to a mean value of nine in the control group (*p* = 0.0011). The mean baseline chronic liver failure consortium acute decompensation (CLIF-C AD) score was 53.9 ± 6.8, indicating an intermediate 3-month mortality risk (7, 28). After initiation of recompensation measures, the CLIF-C AD score reduced slightly to a mean value of 51.3 ± 6.9 on day 3 and to 51.05 ± 8.0 on day 5.

The baseline characteristics of the study population and the control group are shown in Table 1.

**Table 1:** Baseline characteristics.

Variable	Study cohort (*n* = 36)	Control group (*n* = 10)	*p*-value
Age, y	60.8 (9.9)	58.5 (3.7)	0.6364
Male:female, *n* (%)	19:17 (52.8%:47.2%)	6:4 (60%:40%)	–
BMI (mean)	25.6 (5.0)	29.6 (6.9)	0.0585
Etiology of liver disease, *n*			
Alcohol	17 (42.7%)	3 (30%)	–
Viral	4 (11.1%)	3 (30%)	–
MASLD	8 (22.2%)	4 (40%)	–
AIH/miscellaneous (PSC/PBC)	7 (19.4%)	0 (0%)	–
Child-Pugh class			–
A	3 (8.3%)	10 (100%)	–
B	19 (52.8%)	0 (0%)	–
C	14 (38.9%)	0 (0%)	–
INR	1.3	1.0	0.0006
Albumin (g/l)	29.5	42.0	0.0001
Bilirubin (mg/dl)	5.3	1.0	0.0851
Creatinine (mg/dl)	1.4	0.8	0.0398
Platelet count (x 10^3^/ μl)	133.6	168.2	0.1937
AST (IU/L)	61.9	37.0	0.0440
ALT (IU/L)	32.5	31.0	0.8540
MELD	15.9 (6.0)	8.8 (4.3)	0.0011
CLIF C AD score			
Baseline	53.9 (6.8)		
Day 3	51.3 (6.9)		
Day 5	51.05 (8.0)		
Esophageal varices (y/*n*)	31/5	3/7	–
small	11 (30.6%)	2 (20%)	–
medium	20 (55.6%)	1 (10%)	–
large	0 (0%)	0 (0%)	–
Decompensating event			
Gastrointestinal bleeding	4 (11.1%)	–	–
Hepatic encephalopathy	8 (22.2%)	–	–
Bacterial infection	7 (19.4%)	–	–
Ascites	12 (33.3%)	–	–
Hepatorenal syndrome	4 (11.1%)	–	–
Hepatic hydrothorax	1 (2.8%)	–	–
LSM, kPa	55.7 (18.2)	23.53 (9.65)	<0.0001
Spleen size, cm	15.5 (2.5)	12.2 (3.4)	0.0016

Overview of the baseline characteristics of cirrhotic patients with acute decompensation and patients with compensated cirrhosis, serving as control group. Continuous variables are presented as means, standard deviation in brackets. Categorical variables are given as frequencies with percentages. BMI, body mass index; MASLD, metabolic-dysfunction associated steatotic liver disease; AIH, autoimmune hepatitis; PSC, primary sclerosing cholangitis; PBC, primary biliary cholangitis; INR, International Normalized Ratio; AST, aspartate aminotransferase; ALT, alanin aminotransferase; MELD, Model of End-stage Liver Disease.

### 3.1 Spleen and liver stiffness measurement

On admission (day 1), the mean spleen stiffness measured by transient elastography was 70.5 kPa ± 18.3 kPa, in patients with acute decompensation, compared to 29.1 kPa ± 11.2 kPa in the control group of patients with compensated cirrhosis (*p* < 0.0001). The mean longitudinal diameter of the spleen was 15.5 ± 2.5 cm in the group of patients with acute decompensation, compared to 12.2 ± 3.4 cm in the control group (*p* = 0.0016). The baseline liver stiffness (LS) was 55.7 kPa ± 18.2 kPa in the group of acute decompensated cirrhosis versus 23.5 ± 9.7 kPa in the control group (*p* < 0.0001).

**Figure 2:**
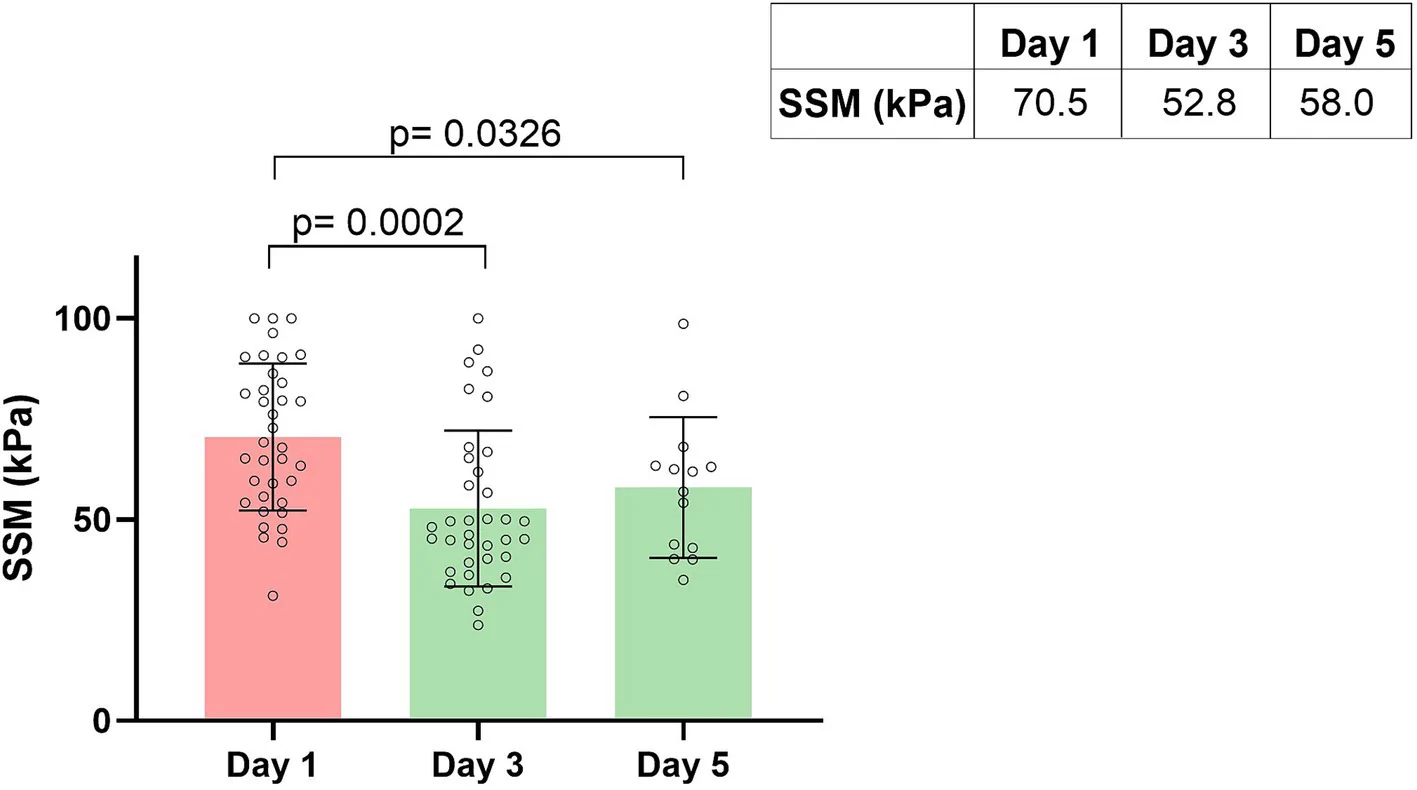
Spleen stiffness measurement (SSM) during recompensation measures. Parameters are given as mean in kPa. SSM, spleen stiffness measurement.

**Figure 3:**
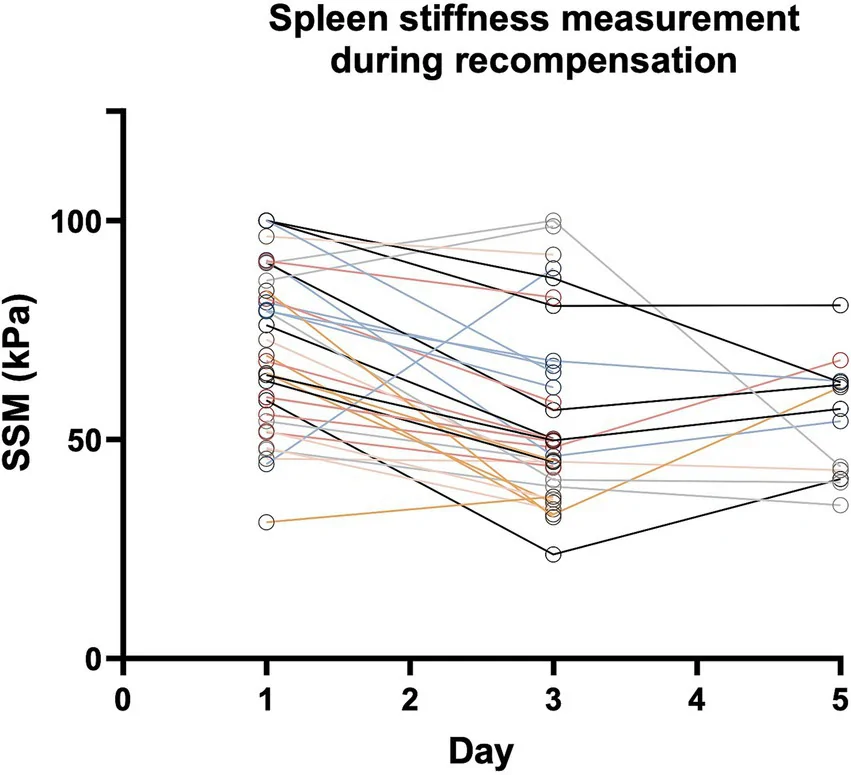
Spleen stiffness measurement (SSM) during recompensation measures. Every Patient is represented by one line. Parameters are given as mean in kPa. Abbreviations: SSM, spleen stiffness measurement.

After recompensation measures had been initiated, a significant reduction in spleen stiffness on day 3 of −18.5 kPa (*p* = 0.0002) was detected in the group of patients with acute decompensation. This corresponds to a relative decrease in spleen stiffness of −21.53% from day 1 to day 3 (Figures 2, 3).

On day 5 of recompensation, spleen stiffness continued to be significantly lower with a decline of −17.63 kPa (*p* = 0.0326), compared to the baseline measurement, corresponding to a relative spleen stiffness reduction of −21.23%. When comparing SSM values from day 3 and day 5, there was no further reduction, instead a plateau-effect (52.77 kPa vs. 57.99 kPa, *p* = 0.4220) could be observed (Figures 2, 3).

Among the 36 patients who received at least two sequential examinations, SSM remained stable in two patients, defined as a reduction of <10% in sequential SSM. Both patients were female and show ascites as decompensating event.

While the majority of patients with acute decompensation showed a reduction in SSM values on day 3 after initiation of recompensation, an increase in SSM on day 3 was observed in three patients indicating recompensation failure. Accordingly, the clinical evaluation of these patients also indicated that the initiated recompensation measures had not been successful. One patient showed weight gain due to rapid recurrent ascites after paracentesis, while another patient experienced acute esophageal variceal bleeding after the initial SSM had been performed. For the third patient, clinical recompensation could be achieved delayed, only at day 5.

In addition, after an initial decrease in spleen stiffness from day 1 to day 3, a renewed increase in spleen stiffness on day 5 was observed in six patients, while three patients showed only a mild increase of <20%. After initial recompensation, all of these patients developed clinical decompensation events such as renal failure (HRS) or recurrent ascites, after a short period of time, indicating that the recompensation measures were inefficient.

When considering the different etiologies patients with cirrhosis due to viral etiology showed the highest reduction (−32.48%) in SSM on day 3, followed by patients with MASLD (−28.64%), alcohol (−20.78%), and finally other etiologies like autoimmune hepatitis, primary sclerosing cholangitis or primary biliary cholangitis (−17.78%).

The control group with compensated cirrhosis showed a spleen stiffness of 29.1 ± 11.2 kPa on day 1 and 32.8 ± 8.6 kPa on day 3, respectively. Thus, there was no significant change between the SSM from day 1 to day 3 (*p* = 0.41) as could be expected in this stable clinical situation. Interestingly, spleen stiffness in patients of the control group was <45 kPa in every single measurement.

## 4 Discussion

The preliminary results of the present pilot study demonstrate that repetitive SSM may be a suitable clinical marker to assess the effectiveness of initiated recompensation measures in cirrhotic patients with acute decompensation.

SSM is an emerging tool in the armamentarium for diagnosing and monitoring patients with CSPH and predicting liver-related outcomes. Since objective measures are essential to standardize and optimize patient management, the use of SSM to assess the effectiveness of recompensation appears extremely useful (29).

To the best of our knowledge, this prospective study is the first analyzing the dynamic changes in spleen stiffness during recompensation in patients with acute decompensated liver cirrhosis.

In contrast to repeated LSM, which did not show a rapid adaptation to treatment measures in patients with acute decompensation (30), repetitive SSM can detect early changes in portal hemodynamics and provide relevant information regarding the effectiveness of initiated recompensation measures. In the present study, a significant reduction in spleen stiffness was observed only 3 days after the introduction of recompensation measures with a subsequent plateau effect. Therefore, in addition to a clinical evaluation, SSM could be a promising diagnostic tool to assess the immediate effectiveness of recompensation in everyday clinical practice. It is a high priority to evaluate the success of initiated recompensation measures timely, in order to identify any non-responders and to be able to adapt treatment concepts that has been initiated early on.

Unfortunately, the definition of “clinical improvement” and hepatic “recompensation” in patients with acutely decompensated liver cirrhosis is inconsistent. In 2021, the Baveno VII consensus conference introduced standardized criteria for the definition of hepatic recompensation, which requires cirrhotic patients achieving (i) a sustained cure with suppression or removal of the underlying etiology of cirrhosis, (ii) a resolution of complications such as ascites and hepatic encephalopathy after discontinuation of diuretic treatment and/or prophylactic therapies, as well as the absence of portal hypertensive bleeding for at least 12 month, and (iii) a stable improvement of liver function, assessed by liver function tests (e.g., serum albumin, bilirubin and INR) (22, 31). Though, a complete and long-lasting resolution of complications is difficult to achieve in clinical practice, since an irreversible alterations of liver structure typically goes along with a compromised liver function and repetitive decompensating events, making continuous treatment measures, e.g., with low dose diuretics, indispensable.

However, while the resolution of a hepatic decompensation following a successful treatment is inevitably linked to an improvement in hepatic function, the Baveno VII criteria neither specify suitable techniques nor define functional parameters or cut-off values that are required for patients to be considered as recompensated.

Non-invasive measurements, such as SSM, could represent such a technique and would be easy to implement in clinical practice. SSM is a promising tool that provides supplementary information quickly, non-invasively and cost-effectively. Accumulating evidence indicates, that SSM appears to outperform liver stiffness as a direct and dynamic marker of CSPH, offering the potential to evaluate improvements in CSPH as an indicator for clinical outcomes (32, 33).

Recently, Colecchia et al. (25) investigate a SSM based predicting model in patients with compensated cirrhosis as predictor for decompensation and observed that the accuracy was at least equivalent to that of invasive HVPG measurement (22). Furthermore, SSM seems to be an accurate non-invasive tool for evaluating the hemodynamic response to non-selective beta blocker (NSBB) therapy, frequently used as prophylaxis in patients with high-risk varices. A SSM reduction by at least 10% (ΔSSM ≥10%) showed excellent accuracy in identifying HVPG responders after NSBB treatment initiation (25).

During acute decompensation SSM increased significantly (34). In a recent study by Meister et al. (34), all patients with acute decompensation had a significant increase in spleen stiffness to values above 39 kPa. Similar findings were also shown in a recently published study from Italy, where the median spleen stiffness in 34 study participants with acute hepatic decompensation was 61 kPa (35).

These results are consistent with the present study, where cirrhotic patients with acute decompensation had a spleen stiffness of 70.5 kPa. In both the Italian and the present study, a specific spleen-dedicated probe (SSM@100 Hz) was used, that allows the most accurate SSM.

However, despite recent advancements in SSM research, it had been unclear so far, how spleen stiffness changes during recompensation. To the best of our knowledge, the present study is the first to demonstrate a role of repetitive SSM in the assessment of the efficacy of recompensating measures. Here, a significant reduction in the SSM values was already observed within the first 3 days after starting recompensation, with a stable plateau of the SSM values afterwards.

The observation that the SSM values of the control group were significantly lower than in the group of patients with acute decompensated cirrhosis, both at baseline and during the short-term follow-up (day 3), supports the conclusion that the presented results can be classified as meaningful and valid.

However, while repeated SSMs during recompensation seems to be suitable to check if the initiated therapeutic measures aimed at recompensation are ineffective, it rens unclear whether a trend toward a “true” reduction in CSPH can be derived from the data. To date, no non-invasive test system (e.g., LSM, SSM, ANTICIPATE model, VITRO score) appears to have the full capacity to replace HVPG measurement as the gold standard for evaluating CSPH and none of the non-invasive procedures or tests currently being discussed seems to have the full potential to adequately monitor short-term dynamics in HVPG, such as HVPG response upon NSBB treatment initiation. For now, this rens the exclusive don of repeated invasive HVPG measurements, and although SSM has shown some promise results, this needs to be further investigated in future studies (24, 36–38).

Furthermore, while repetitive SSM measurements enable early re-assessment of recompensation measures.

The preliminary results of this pilot study cannot provide a conclusive answer whether the recompensation measures ren effective in the long-term, beyond day 5, and cannot estimate the overall risk of recurrent decompensation in the follow-up. To address this question, further studies with a long-term follow-up and repetitive SSM are required.

Interestingly, the extent of spleen stiffness reduction in the present study varied depending on the underlying etiology of cirrhosis. Patients with an acute decompensated cirrhosis due to a viral etiology showed the highest reduction (−32.48%) in SSM on day 3, followed by patients with MASLD (−28.64%), and alcohol (−20.78%), while the underlying mechanism ren elusive. It seems possible that the observed differences are related to the varying underlying pathogenic mechanism and pathophenotypes of portal hypertension of the respective etiologies. Different liver diseases such as viral-, metabolic-or alcohol-related liver disease show different structural changes of the liver architecture along with different alterations in the hepatic microcirculatory system on the one hand and different degrees of systemic inflammation across acute decompensation on the other hand (39–50). Both aspects are closely interrelated and have a significant influence on portal hemodynamics, which are reflected in changes of spleen stiffness.

While liver stiffness varies depending on the underlying etiology, as recently demonstrated by Jachs et al. (16) in a study of 420 patients showing that patients with alcoholic liver disease (ALD) had higher LSM than patients with viral etiology or MASLD, there are no comprehensive data on the changes in spleen stiffness depending on the etiology of the underlying liver disease so far (16).

Due to the small number of cases in the individual subgroups, no final conclusions regarding the different SSM of the varying etiologies can be drawn from the preliminary findings of the present pilot study. However, comprehensive follow-up studies with larger numbers of patients of different etiologies are urgently required to evaluate the extent to which the SSM patterns differs according to the underlying etiology.

The main limitations of our study derive from the monocentric design and its small selective study cohort following the large number of strict exclusion criteria. However, the underlying study was a pilot project that was initially intended to test the feasibility and usefulness of repetitive SSM in the clinical management of patients with acute decompensation. Another limitation of the study is that only a small proportion of patients received SSM on day 5, since several patients were discharged beforehand. A further limitation is that the recompensation measures applied were different, depending on the type of decompensation. In future studies with larger cohorts, the SSM should be analyzed in dependence of the initiated recompensation measure. Finally, regression to the mean, a phenomenon well known from repeated measurements of physiologic parameters needs to be considered when interpreting the observed changes in SSM (51).

In conclusion, repetitive SSM in patients with acute decompensation seems to be a promising non-invasive method to assess recompensation measures that deserves to be studied in larger patient populations.

## Data Availability

The original contributions presented in the study are included in the article/supplementary material, further inquiries can be directed to the corresponding authors.
